# Mercy Hospital Surgical Report

**Published:** 1879-11

**Authors:** E. C. Helm

**Affiliations:** House Surgeon


### NOTES FROM HOSPITAL PRACTICE.

### ARTICLE X.

### MERCY HOSPITAL SURGICAL REPORT. IN THE SERVICE OF PROF. EDMUND ANDREWS. REPORTED BY E. C. HELM, M.D., House Surgeon.

*Recurrent Tumor of the Knee.*

Miss B. G., aged 22, entered the hospital Sept. 13, 1878, with a swelling just below the knee, the leg at that point being one-third larger than normal. The tumor was soft, semi-fluctuating, and without any well defined border.

Severe pains radiated from this region upward to the thigh, and downward to the foot, being especially severe at night.

Sept. 16th.—Gave ether and punctured the swelling with an aspirating needle, but obtained no pus. Then a free incision was made, and the tumor found to consist of small nodules. These nodules were formed of fat and white fibrous tissue—the latter predominating.

There being no well defined border to the tumor, it was not enucleated, but the wound was dressed with simple cerate.

Sept. 24.—The wound having rapidly healed, and there having been no return of the pain, she went home to-day.

Feb. 28th, 1879.—She returned to-day with the tumors rather larger than when she first entered, and the pain is now excessive.

Directed, quin. sulph., potas. iod. internally, an anodyne liniment externally.

March 11th.—The pain continuing, ether was given and a transverse incision one decimeter in length made just below the knee and the tumor removed. The cutaneous branches of the internal saphenous nerve were found to have been involved in the hypertrophied connective tissue. The wound was then dressed, using carbolized zinc wash and carbolized oil covering.

March 20.—Patient went home to-day ; the incision has nearly united, and there is no pain.

Sept. 3d, 1879.—The patient has sent word that she has entirely recovered.

*Recurrent Frontal Abscess.*

E. B. S., aged 42, entered the hospital April 22, 1879, with the following history :

For the past sixteen years he has had paroxysms of intense neuralgic headache, occurring at quite regular intervals of from three to four weeks. The headache was usually attended with flexion of the hands and fingers, and of the toes as well. There was a sensation as if the extensor muscles were "asleep." The same sensation was felt in the muscles of mastication, accompanied with slight rigidity of the jaws. These deranged sensations and motions disappeared with the headache, only to recur with the next paroxysm.

Six years ago he was struck in the forehead, just above the bridge of the nose by the thumb-piece of the hammer of a gun, the thumb-piece reaching the bone. The resulting wound healed by first intention. Nothing new occurred for eighteen months, the periodical headaches continuing as before.

After the lapse of eighteen months, at the time the headache should occur, he noticed that the pain became localized, being upon the right side of the forehead, just above the external angular process of the orbit. The pain was accompanied by swelling, but no fluctuation. The pain soon became intense, of a throbbing character, and accompanied by considerable fever. The swelling gradually extended forward until the seat of the old wound, already alluded to, was reached, when it (the swelling) became softer and more fluctuating. The course of the affection would now vary, about every second time the swelling would subside, the pain and fever diminish, and the patient would be relieved, no noticable discharge having passed through the nose.

In the intermediate paroxysms, the swelling opens spontaneously, at the old wound, evacuates its contents, consisting mainly of pus, remains open a few days, discharging slightly, and then closes up. The patient would be free from pain for the next three weeks.

These paroxysms usually lasted about one week.

April 25th, 1879.—Ether was administered, the cicatrix excised, and a canal found leading down to the frontal sinus. This sinus contained a few drops of thick yellow pus.

A bent probe could be passed into the nasal fossa by the use of some force and the exercise of considerable patience. No dead bone could be found. After cleansing the wound, fluid was injected into the nose, and it (the wound) was dressed openly.

May 17th.—Fluid has been injected into the nose through the wound daily to ensure the passage remaining open.

Prof. Andrews to-day, after repeated, careful efforts succeeded in finding a small scale of dead bone about one centimeter in diameter, at or near the seat of the lachrymal bone. The bone could be felt only by pressing a sharply bent probe into the nose through the wound.

The patient now became anxious to return home, and as the bone could not be removed without injuring his features, he was advised to go home, and to inject a solution of

| | | |
|---|---|---|
| Acid. carbol. | | 1 |
| Acid. muriat. dil. | 1 | |
| Sodii chlor. | 1 | 2 |
| Aquæ. | 50 | |

morning and evening, with the view of dissolving the bone.

The quantity of dilute hydrochloric acid was to be slowly increased until doubled, and the injection was to be used as long as there was any discharge.

May 19th.—The patient went home to-day.

Aug. 10th.—Letters received at intervals state that the patient is slowly improving, having had no return of his old enemy, the sick-headache.

Sept. 2.—Another letter just received states that he considers himself about well, that the external opening has closed, and there has been no return of the paroxysms of headache.

As to whether the opening into the nose from the frontal sinus remained patulous or not, he is unable to state, but thinks it is open, as there is no re-accumulation of fluid in the frontal sinus.

*Malignant Sarcoma.*

J. S. S., aged 48, came to the hospital for an operation Oct. 7th, 1878.

He had a tumor, .07 by .05, beneath the sterno-cleido mastoid muscle, which is not tender on pressure, nor has he had any shooting pains.

There are about a dozen small tumors surrounding the larger one, which are evidently enlarged lymphatic glands.

Ether was administered, and the tumors removed. The large tumor was in the inferior carotid triangle, and was also partly under the sterno-cleido-mastoidius.

Oct 10th.—Tumor examined microscopically by Prof. Andrews. It showed a round-celled sarcoma, there being only a few scattered fibers, and nothing like a stroma being seen.

The mass was almost a continuous area of round cells. It had a capsule, from which it could easily be peeled, and had a gray appearance. The smaller tumors presented the same characteristics.

Oct. 11th, the patient has gone home, and his family physician advised to put him upon a course of arsenic treatment.

May 19th.—Patient has again returned for operation, the tumor having returned.

It is as large as a hen's egg, and is posterior to the seat of the original tumor, being entirely posterior to the sterno-cleido-mastoideus, the anterior line of the tumor being limited by the cicatrix formed by the first operation.

Ether was given and the tumor removed.

The patient remained a few days in the city, during which the wound was dressed, and went home May 24th.

July 21st.—Returned to-day with a tumor in the seat occupied by the first tumor. The present one is about the size of a walnut. Ether was given and the main tumor removed. Many small tumors were found in close relation with the external and common carotids.

These small tumors extended down into the thorax, surrounding the subclavian artery, so that it was impossible to remove them all. Those that could be reached were carefully dissected from their connection with the carotids. It was found necessary to ligate the external jugular vein, the ascending cervical and transversus colli arteries, and various smaller arteries. The wound was then dressed. In half an hour the hæmorrhage returned and was excessive, the man losing over a litre of blood in less than two minutes.

Firm pressure was then made, which partially controlled it. The dressings and sutures were then removed, and the wound opened. The impression had been that the ligature from the external jugular had slipped, but that was found to be in place, as were the other ligatures. Nothing but a general oozing could be found. The source of the hæmorrhage was therefore supposed to be the oozing which did not find an outlet until it had forced one by its collection and pressure.

The wound was re-closed, but in an hour had to be re-opened, as the oozing was increasing. After being exposed to the air for half an hour it was again closed, after which there was no more hæmorrhage.

July 23. Doing well. Went home to-day.

Sept. 1. Letters received state the tumors are returning, but he was advised not to undergo another operation as they have already extended into the thorax beyond the surgeon's reach.

*Fungus Hœmatodes and Melanotic Carcinoma.*

C. F. K., entered the hospital, May 31st, 1879, with history as follows:

About two years ago he noticed a wart on the outside of the right arm, near the shoulder which soon began to grow.

It was then bitten off by a horse, but soon returned. It then began to grow rapidly and to assume the form and color of a fungus hæmatodes, being globular in form, and as large as a good sized hickory-nut. It has a rather large pedicle, and bleeds easily. He now began bandaging it tightly, so that when he entered the hospital it was shaped like a mushroom. There is also a multiple tumor in the right axilla as large as an orange.

On the back of the left arm, just above the elbow there is a small subcutaneous tumor, and in the substance of the gluteus maximus muscle of the left side is another tumor about the size of a walnut.

June 2.—Ether was administered and the four tumors removed.

The one upon the right arm, as before, mentioned, was a well marked fungus hæmatodes. The one in the right axilla involved the right axillary glands, and in fact seemed to be composed mainly of them. It was of a dense, firm character, and of a whitish color, rather pearly.

Both tumors on the left side were very dark, almost black, good examples of melanotic tumors.

The tumors were subsequently examined with the microscope and found to be carcinomatous.

June 10th.—All the wounds have united by first intention except the one under the arm, which is slowly granulating.

June 11th.—Had several chills this forenoon.

At 3:30 p. m. the temperature was 39.7, while the pulse was but slightly accelerated.

There was no appearance of eyrsipelas, still it was deemed best to give full dose of cinchonidia and tr. iron.

June 12th.—There is a slight diffused redness around the axillary wound.

The temperature ranges from 39 to 40.6, with the pulse still below 100. Directed a wash of

| | | |
|---|---|---|
| Acid. carbol. | 1 | 3 |
| Zinc chlorid. | | |
| Aquæ | 50 | |

to be kept constantly applied.

June 13th.—The erysipelas is slowly spreading. The fever has subsided. Applied a solution of nitrate of silver,

| | |
|---|---|
| Argt. nit. | 2 |
| Aquæ | 50 |

by means of a camel's hair brush around the border of the erysypelas, but it did not check it.

June 16th.—The erysipelas, which is purely cutaneous, now involves one-half of the trunk.

June 20.—Erysipelas has disappeared.

June 25.—Patient left contrary to advice. The wound is slowly granulating.

Aug. 29th.—A letter from the patient states that the cut under the arm has nearly healed, having been delayed by sickness (the sickness being purely medical and not surgical) but now he is almost entirely well with no signs of any more tumors.

*Excision of Shoulder Joint for Necrosis.*

S. R., aged 33, entered the hospital May 27th, 1879, with the following history:

While in the army (during the civil war) he was struck in the left shoulder by a fragment of a spent shell, which inflicted but a slight bruise. However, the injury was followed by a slight fever, lasting but a few days, after which he apparently recovered entirely.

He had no more trouble with the shoulder until November, 1878, then, while carrying his arms full of stove-wood, his left arm suddenly and completely lost its power.

The shoulder soon became somewhat swollen and very painful, the pain becoming agonizing and not controlled by excessive doses of morphia. The family physician made an incision at the apex of the shoulder, and a very small amount of pus escaped. Another opening was spontaneously made over the spine of the scapula near its center.

There was, when entering the hospital, an entire absence of sensation over the spine of the scapula, and for a distance of five centimeters on each side, while over the apex of the shoulder there was moderate tenderness. A probe could be passed from one to the other of these two openings. The discharge was exceedingly slight, and semi-purulent in character. No dead bone could be found, though in the posterior fistula the probe grated upon fine sand-like particles which seemed to be imbedded in the substance of the muscles lining the fistula. The patient could not afford to lose much time, so before an accurate diagnosis was reached, ether was given, and an exploratory incision made from the tip of the acromion process through the deltoid nearly to its insertion. The head of the humerus being found honeycombed, was removed at the surgical neck by means of the chain saw. The tip of the acromion process was removed. The incision was then carried backward along the spine of the scapula for one-half its length, and a small triangular incision was made at the inner end of the scapular spine.

Through these incisions the spine of the scapula could be felt and was found to be soft and friable, and it was easily gouged out, indeed, a large part could be removed by the finger nail. This would thus seem to be one of those rare cases of necrosis of the shoulder in which the disease *originated* in the spine of the scapula.

Drainage tubes were put in, and the long incision was brought in apposition by sutures, over which broad and long adhesive straps were applied. The hæmorrhage was slight. Directed cinchonid. .015 and tr. ferri chlorid. .4 four times a day.

July 6th.—There has been considerable oozing, in fact so much that although dressed twice a day, the dressings have been thoroughly saturated. The pulse has ranged from 100 to 116, and the temperature from 37.8 to 39.2, being rather higher in the evening than in the morning.

July 8th.—Oozing has ceased; pulse and temperature but little above normal.

July 9th.—Has been up several hours; eats and sleeps well; temperature and pulse about normal. Drainage tubes removed. Wound closing rapidly with little discharge.

July 11th.—Has slight fever and is rather restless.

July 12th.—Fever increasing slowly; he does not sleep well; no appetite, and is very restless; still granulation is going on rapidly.

July 13th, 8 a. m.—Pulse, 100; temperature, 39.2.

Directed

| | |
|---|---|
| Acidi salicylici | 15 |
| Sodii bicarb | 10 |
| Tr. opii camph | |
| Aquæ *āā* | 40 |

and directed him to take 4 gms. every three hours — day and night — alternating with cinchonid. .03 and tr. ferri cl. .4.

In spite of this treatment the fever continued to rise. Below will be given the range for two days.

July 13—1 p. m.—Pulse, 108; temperature, 40.4; 3 p. m. Pulse, 108; temperature, 40.4; 5 p. m.—Pulse, 110; temperature, 40.5; 7:30 p. m.—106; temperature, 40.3; 9 p. m.—Pulse, 100; temperature, 40.

July 14th—7:30 a. m.—Pulse, 106; temperature, 40; 11 a. m.—Pulse, 110; temperature, 40.4; 1 p. m.—Pulse, 110; temperature, 40.5; 3.30 p. m.—Pulse, 116; temperature, 40.7; 5.30 p. m.—Pulse, 108; temperature, 40.4; 9 p. m.—Pulse, 100; temperature, 38.3.

Dropped the tr. of iron as he was becoming nauseated, and gave dilute hydrochloric acid in full doses; also pushed the cinchonidia and salicylic acid.

July 15.—The pulse and temperature have ranged as on yesterday, but have been a shade higher. Notwithstanding the continuous high temperature the wound looks well; granulation is good, and there is no trace of erysipelas; was slightly delirious last night.

July 16.—The temperature ranged from 40. to 40.6 all night. Directed the night and morning doses of cinchonidia sulph. to be replaced by quin. sulph. There is this evening a faint erysipelatous redness around the wound. Directed the carbolized zinc wash to be kept constantly applied, and increased the dose of hydrochloric acid.

July 17th.—The redness has spread down the arm. Painted a belt, .06 in width, around the reddened space, of a solution of nitrate of silver,

| | |
|---|---|
| Argt. nit | 2 |
| Aquæ | 50 |

which appeared to limit its spread on the body, but had no effect on the arm, as the erysipelas spread to the elbow.

Notwithstanding his erysipelas, his appetite has increased, he having eaten almost nothing for the past week until to-night when he ate quite heartily.

July 18—20th.—About the same.

July 21st.—Erysipelas fading; sleeps well; appetite good.

July 26th.—Walks around the ward and feels quite well. The wound is closed except at the points where the drainage tubes had been inserted.

July 27th.—Went home to-day.

Sep. 2d.—A letter just received states that the wound rapidly closed up after leaving the hospital, but that the arm, while being of considerable use, is far from being strong.

---

MEDICAL REPORT. Service of DRS. N. S. and F. H. DAVIS.

Reported by H. M. STARKEY, M. D., House Physician.

*Case 1.*—J. S., German, age 40, bachelor, peddler. Entered hospital April 19, 1879.

The only history obtainable from friends is as follows: Patient has always been very healthy, and retired last night apparently in perfect health. Was found in bed at half past six this morning, breathing heavily and frothing from the mouth. Within a few minutes he had a slight convulsive seizure, which stiffened most of the muscles without producing decided spasm. Was taken to the hospital two blocks distant, and first seen by the house doctor about half past seven a.m.

